# Timing of ergogenic aids and micronutrients on muscle and exercise performance

**DOI:** 10.1186/s12970-019-0304-9

**Published:** 2019-09-02

**Authors:** Richard A. Stecker, Patrick S. Harty, Andrew R. Jagim, Darren G. Candow, Chad M. Kerksick

**Affiliations:** 1 Exercise and Performance Nutrition Laboratory, School of Health Sciences, Lindenwood University, St. Charles, MO 63301 USA; 2 Human Performance Lab, Sports Medicine, Mayo Clinic Health System, Onalaska, WI USA; 3 Faculty of Kinesiology and Health Studies, University of Regina, Regina, SK S4S 0A2 Canada

**Keywords:** Nutrient timing, Caffeine, Creatine, Supplements, Performance, Strength, Power, Beta-alanine

## Abstract

The timing of macronutrient ingestion in relation to exercise is a purported strategy to augment muscle accretion, muscle and athletic performance, and recovery. To date, the majority of macronutrient nutrient timing research has focused on carbohydrate and protein intake. However, emerging research suggests that the strategic ingestion of various ergogenic aids and micronutrients may also have beneficial effects. Therefore, the purpose of this narrative review is to critically evaluate and summarize the available literature examining the timing of ergogenic aids (caffeine, creatine, nitrates, sodium bicarbonate, beta-alanine) and micronutrients (iron, calcium) on muscle adaptations and exercise performance. In summary, preliminary data is available to indicate the timing of caffeine, nitrates, and creatine monohydrate may impact outcomes such as exercise performance, strength gains and other exercise training adaptations. Furthermore, data is available to suggest that timing the administration of beta-alanine and sodium bicarbonate may help to minimize known untoward adverse events while maintaining potential ergogenic outcomes. Finally, limited data indicates that timed ingestion of calcium and iron may help with the uptake and metabolism of these nutrients. While encouraging, much more research is needed to better understand how timed administration of these nutrients and others may impact performance, health, or other exercise training outcomes.

## Background

Nutrient timing involves the purposeful consumption of nutrients and related ergogenic aids at specific time points to potentially maximize performance, augment training adaptations, and promote recovery [1]. To date, the majority of nutrient timing research has examined the efficacy of two macronutrients: carbohydrates and proteins [2]. However, a small, but emerging body of literature indicates that the strategic ingestion of caffeine, creatine, dietary nitrates, sodium bicarbonate, beta alanine, iron, and calcium may influence muscle and exercise performance. Thus, the purpose of this narrative review is to briefly and comprehensively summarize the body of literature investigating acute and chronic supplement timing strategies of micronutrients and non-nutrients and to offer potential directions for future timing research in this area.

### Timing strategies to improve performance

#### Caffeine

Caffeine is a trimethylxanthine which is catabolized by the P450 cytochrome system in the liver to three dimethylxanthines: theophylline, theobromine, and paraxanthine (for review see Graham et al. [3]). Caffeine may influence muscle and exercise performance by acting as an adenosine receptor antagonist [4–6] or by influencing phosphodiesterase [7], and excitation-contraction coupling [3, 5]. Acute ingestion of 3–6 mg per kilogram (mg/kg) caffeine prior to endurance exercise has been shown to favorably impact fat utilization, reduce perceptions of fatigue, increase one’s drive to exercise, and enhance performance [8–10]. Similarly, studies employing resistance-training have reported mixed results following pre-exercise caffeine ingestion, with some studies reporting significant increases in force production and muscular endurance [11, 12] while others have failed to detect such changes [13, 14]. In a recent meta-analysis, Grgic et al. [15] examined the effects of caffeine supplementation in 149 participants across 10 studies and concluded that caffeine supplementation significantly improved muscle strength (standardized mean difference: 0.20, 95% confidence interval [0.03, 0.36], *p* = 0.023). Polito et al. [16] performed a meta-analysis on 17 studies consisting of 227 men and 21 women to determine the effects of caffeine on muscle performance. Results showed that caffeine supplementation improved muscle endurance and performance (Effect sizes: 0.29–0.48, *p* < 0.01). Because plasma caffeine levels typically peak within 60 min of ingestion [8, 17], the attention given to the timing of caffeine consumption relative to exercise is logical. However, it is important to note that differences in mode of caffeine administration (e.g. caffeine gum, capsule, or aqueous solution) [18], lack of diverse exercise interventions, and underlying genetic factors that influence caffeine metabolism make conclusions derived from available caffeine timing studies somewhat limited in scope [19]. Nevertheless, several recent investigations comparing the effects of pre-exercise and intra-exercise caffeine ingestion on exercise performance have provided valuable information for individuals seeking to maximize the ergogenic effects of caffeine.

To date, all caffeine timing studies have exclusively utilized cycle exercise models. Bell and McLellan [20] showed that well-trained individuals who consumed caffeine (5 mg/kg) 1, 3, or 6 h prior to performing cycling exercise to fatigue experienced a significant (*p* < 0.05) increase in exercise time-to-exhaustion only after 1 and 3 h pre-exercise ingestion of the substance. Cox and colleagues [21] likewise compared the effects of several protocols of caffeine ingestion on cycle time-trial performance in highly-trained cyclists after completing a two-hour bout of steady-state cycling at 70% peak oxygen consumption (VO_2_peak). Participants consumed 6 mg/kg caffeine in capsule form 1 h prior to the steady-state bout or consumed six doses of 1 mg/kg caffeine every 20 min during the steady-state bout before completing a time-to-exhaustion bout of cycling at 70% VO_2peak_. Caffeine ingestion improved time-trial performance relative to placebo in both conditions with no differences in performance between groups, though time trial performance was only significantly greater (*p* = 0.04) than placebo following pre-exercise ingestion of the substance. Similarly, Conway and colleagues [22], found no added ergogenic effect of a divided caffeine dose compared to a single caffeine-matched dose (6 mg/kg) administered via capsules 1 h prior to cycle exercise. Finally, it appears that intra-exercise administration of caffeine as part of a rehydration strategy may be an effective method to improve subsequent performance. Talanian and Spriet [23] administered 100 mg or 200 mg caffeine as part of a carbohydrate-electrolyte solution to cyclists after 80 min of a 120-min steady state cycling bout which was immediately followed by an additional 120 min cycling time trial. Unsurprisingly, both caffeine conditions significantly (*p* < 0.05) improved time trial performance compared to placebo, though the 200 mg dose of caffeine improved time trial performance to a greater extent than the 100 mg dose. Taken together, these results suggest that intra-exercise caffeine ingestion during prolonged cycle exercise may be equally effective relative to pre-exercise consumption.

Two studies have investigated the timing implications associated with caffeinated chewing gum [24, 25], which is absorbed at a faster rate than capsules [26]. Ryan and colleagues [25] investigated the effects of caffeinated chewing gum (200 mg) administered 35 and 5 min prior to a cycling time-to-exhaustion test at 85% VO_2_max (maximal oxygen consumption) and again 15 min post-exercise. Caffeine had no effect on exercise performance, possibly due to the low caffeine dosage utilized. In a subsequent study [24], the researchers administered caffeinated chewing gum (300 mg) to male cyclists at 2 h, 1 h, and 5 min prior to a cycling time trial. The authors reported a significant improvement (*p* = 0.023) in time trial performance only when caffeine was dispensed immediately prior to exercise (38.7 ± 1.2 min) when compared to 60 min pre-exercise (41.8 ± 2.6 min) and 2 h pre-exercise (42.6 ± 2.2 min) [24]. Results across studies suggest that caffeine chewing gum (300 mg) immediately before aerobic exercise to exhaustion may exert a small ergogenic outcome. However, more studies in a wider variety of exercise modalities are required before results can be generalized further. In addition, the varying impact of different modes of caffeine administration must be quantified, as differences in speed of absorption and bioavailability [26] may dramatically alter the efficacy of a given timing protocol.

#### Dietary nitrates

In recent years, dietary nitrates have drastically increased in popularity as a large body of peer-reviewed studies have documented their efficacy to improve performance during endurance [27] and intermittent [28] exercise. Nitrates are found in leafy greens such as spinach, lettuce, and celery as well as in root vegetables such as beetroot [27]. When ingested, dietary nitrate (NO_3_^−^) is reduced to nitrite (NO_2_^−^) by bacteria in the oral cavity and then to nitric oxide (NO) in the stomach, though some nitrite has been shown to enter systemic circulation [29]. NO may improve exercise performance by enhancing blood flow and muscular contractility [29] and reducing the oxygen cost associated with aerobic exercise [30]. To date, many of the investigations that have employed dietary nitrate consumption have utilized a prophylactic supplementation period of 3–6 days, though researchers examining the effects of acute dietary nitrate consumption have often administered the supplement approximately 2–3 h prior to exercise [27]. However, limited information exists regarding the timing of acute nitrate intake. Hoon et al. [31] recently compared the impact of three nitrate timing strategies in national-level cyclists who performed two separate bouts of 4 min time trials separated by 75 min of rest. In a counter-balanced, double-blind, crossover fashion, participants consumed three combinations of beetroot juice or placebo at 150 min and 75 min prior to the first time trial. The combinations included the following: beetroot juice administered 150 min prior to the first time trial (placebo was ingested at 75 min), beetroot juice administered 75 min prior to the first time trial (placebo was ingested at 150 min), and beetroot juice administered at both time points. However, dietary nitrate supplementation (irrespective of timing) did not statistically improve time-trial performance during the first trial, and the supplemental conditions may have slightly impaired performance during the second time trial compared to placebo. The authors noted that nitrates from other dietary sources were not restricted during the study, which may have reduced the impact of nitrate supplementation compared to other studies which completely limited dietary nitrate consumption in all participants during the study period. Clearly, further research is required in this nascent area before clear conclusions can be made (Table 1).

**Table 1:** Timing implications of acute supplement administration

Documented evidence of timing benefit				
Nutrient/Dietary supplement	Mechanism of action	Purported benefit	Recommended dosing protocol	Timing-related references
Caffeine	Adenosine receptor antagonist	↑ fat utilization, ↑ mental drive, ↑ performance, ↓ perceptions of fatigue, ↑ force production, ↑ muscular endurance	Time: 2 h – 0 h Absolute dose: 100 mg – 300 mg Relative dose: 3–6 mg/kg body mass	[20–25]
Nitrates	↑ nitric oxide ↑ blood flow ↑ muscular contractility ↓ O_2_ cost during aerobic exercise	↑ endurance ↑ intermittent exercise	Time: 2–3 h prior Dose: Manufacturer recommendations	[31]

*h* hours, *mg* milligrams, *mg/kg* milligrams per kilogram of body mass, *O*_*2*_ oxygen

#### Creatine monohydrate

Creatine is one of the most popular as well as the most scientifically examined dietary supplement to date. In this respect, creatine supplementation has been repeatedly demonstrated to improve high-intensity exercise capacity and increase muscle mass and muscle performance in conjunction with resistance training, by influencing high-energy phosphate metabolism, cellular hydration status, muscle protein kinetics, satellite cells, anabolic growth factors, and inflammation [32, 33].

The timing of creatine ingestion may be an important strategy to enhance the physiological adaptation from resistance training. For example, Cribb and Hayes [34] provided matched groups of resistance-trained males with a supplement containing an identical dose of protein, carbohydrate, and creatine monohydrate throughout a structured 10-week resistance training period. When the combination of nutrients was provided in close temporal proximity to each workout (vs. in the morning and evenings), significant increases in strength (*p* < 0.05) and muscle mass (*p* < 0.05) were reported. Most interestingly, significantly greater intramuscular levels of phosphocreatine and creatine were found in the group that provided creatine close to each workout, suggesting that, in addition to promoting positive training adaptations, timing may favorably influence creatine uptake [34]. Later, Antonio and Ciccone [35] published a study directly examining the impact of timed administration of creatine monohydrate. Nineteen recreational male bodybuilders were randomly assigned to receive 5 g of creatine monohydrate either immediately before or immediately after exercise during a structured, four-week resistance training program. While no statistical significance thresholds (*p* > 0.05) were crossed, a magnitude-based inference approach suggested that post-exercise administration might afford more beneficial changes in fat free mass, fat mass, and upper-body strength compared to pre-exercise ingestion. Candow [36] assigned 22 untrained older adults into two groups in a randomized, double-blind fashion: one that received creatine immediately prior to and another group that received creatine immediately after their workouts. Both groups received the same creatine dose (0.1 g∙kg∙day^− 1^) and trained three times per week for 12 weeks. However, no differences in lean mass, muscle thickness and muscle strength were found between the groups. Unfortunately, the aforementioned studies did not include a placebo (control) group. To overcome this limitation and to directly compared the effects of pre-exercise versus post-exercise creatine supplementation, Candow [37] investigated the effects of creatine (0.1 g∙kg^− 1^) immediately before or immediately after resistance training (3 workouts per week), compared to placebo, for 32 weeks in aging adults. Results showed that pre-exercise and post-exercise creatine supplementation increased muscle strength compared to placebo (*p* < 0.025), but there were no differences in strength gain relative to the timing of when creatine was provided. Interestingly, only post-exercise creatine led to greater gains in lean tissue mass compared to placebo. The disparate conclusions seen among the creatine studies are likely due to factors such as small numbers of study participants, a mixed gender cohort [38], or the inclusion of ‘responders’ and ‘non-responders’ [39] in the study protocol. While it is difficult to compare results across studies which use different methodologies, it appears that pre-exercise and post-exercise creatine supplementation are effective strategies to increase muscle mass and strength, with potentially greater muscle accretion benefits from post-exercise creatine.

#### Iron

Iron is an essential mineral which is vital for DNA synthesis, electron transport within the cell, and oxygen transportation to tissues via hemoglobin, as roughly 70% of the body’s iron is bound to hemoglobin in red blood cells [40]. However, several investigations have shown that regular aerobic exercise may decrease iron stores in the body [41–43]. Iron supplementation has been used to help increase aerobic performance through the restoration of hemoglobin concentrations and subsequently improve oxygen carrying capacity within the body [44]. However, iron supplementation does not appear to elicit an ergogenic effect on aerobic exercise performance unless the individual is iron-depleted or has anemia, particularly in females [45, 46]. Nevertheless, the development of strategies to improve iron status may be of interest to researchers and those who work with at-risk populations.

Some investigative work has been completed to determine if the timing of feeding in reference to exercise may favorably impact iron status. Initial interest directed towards this research question was generated from 2002 findings by Matsuo and colleagues [47] which showed that an increase in heme biosynthesis occurs following resistance-style exercise in iron-deficient rats. The researchers hypothesized that post-exercise feeding may even further potentiate heme production and provided two groups of 4-week old male rats similar, iron-deficient feed either immediately after or 4 h after performing climbing exercise three times per week over a three-week period. Plasma iron was significantly elevated (*p* < 0.05) after climbing in only the group which received immediate post-exercise feeding, while hematocrit and hemoglobin levels were similar between groups pre-to-post exercise [48]. The authors subsequently concluded that post-exercise meal timing of iron may increase plasma iron levels but has no effect on hematocrit or hemoglobin concentrations in the blood. However, a key consideration in relation to the potential impact of timed administration of various micronutrients is how the nutrient in question is metabolized and stored within the body. For instance, many vitamins and minerals build up in tissues over time after chronic consumption. Consequently, daily timed administration may exert little influence on certain, but not necessarily all outcomes of interest, such as blood cell counts, electrolyte balance, enzyme activity, metabolic activity, and performance. Therefore, more research is needed to better understand if timed administration of iron or other micronutrients can make a measurable impact on chosen outcomes.

#### Calcium

Calcium (Ca^2+^) is a mineral commonly consumed from various dietary sources such as dairy, leafy green vegetables, and beans [49, 50]. Approximately 99% of calcium is stored in the skeletal system, while the remaining is present in locations such as muscle cells [51]. While some investigators have suggested that calcium supplementation may not possess ergogenic potential due to the body’s ability to utilize the vast depot of calcium stores located in the skeletal system, Williams [51] and Kreider [44] have asserted that calcium supplementation may be beneficial for athletes with an inadequate dietary intake. One of the primary actions of calcium is the facilitation of skeletal muscle contraction [52]. Calcium has also been shown to help maintain bone mass in athletes susceptible to premature osteoporosis as well as improve exercise capacity in calcium deficient athletes [44]. Supplemental calcium also helps blunt the effects of increased levels of parathyroid hormone, which is known to be a potent stimulator of bone resorption [53]. Due to the important actions of calcium, it is evident that more information is needed to better understand if the timing of calcium intake may favorably impact performance or health-related outcomes.

Non-weight bearing activities of a prolonged nature, such as cycling, have been documented to have a negative effect on bone mineral density over time. Barry et al. [53] compared the impact of two different timing strategies of calcium supplementation on calcium homeostasis following cycling exercise. Using a double-blind, crossover design, 20 trained male cyclists completed an intense 35-km (km) cycling time trial. Participants consumed a beverage containing one total gram of calcium either 20 min prior to exercise or in equal doses ingested every 15 min during the one-hour cycling bout. A placebo beverage was provided during the alternate consumption period for each timing condition and the results were compared against a placebo-only condition. The authors found that providing calcium prior to exercise significantly diminished (*p* = 0.04) the expected increase in parathyroid hormone provoked by exercise, although a similar outcome appeared to be occurring when calcium was provided throughout the exercise bout. Due to the well-characterized increase in parathyroid hormone secondary to even minor decreases in serum calcium levels, the blunting of parathyroid hormone indicates an improved maintenance of serum calcium, an effect that was at least partially modulated by the timing of supplemental calcium [53]. A follow-up study randomly assigned 52 competitive male cyclists to groups who consumed 1 g of calcium and 1000 International Units (IU) of vitamin D either 30 min before or 1 h after a strenuous 35-km cycling time trial. When supplements were provided before exercise, the typically-observed post-exercise decrease in serum ionized calcium was significantly reduced. Additionally, a trend for decreased parathyroid hormone levels after exercise was observed in the pre-exercise calcium condition [54]. The same research group completed another study that further examined the impact of calcium timing on calcium homeostasis [55]. As part of two separate experiments, the researchers recruited 50 to 75-year-old women to perform 60 min of treadmill walking at 75–80% peak oxygen consumption. Throughout the first study, ten subjects consumed a calcium-fortified beverage or placebo in equal quantities every 15 min, starting 1 h before exercise and continuing throughout the hour-long exercise bout to deliver a total dose of 1 g of calcium. The second experiment required a group of 23 subjects (healthy post-menopausal women; 50–75 years of age) to consume equivalent amounts of calcium or placebo 15 min prior to exercise and throughout the exercise session in a fashion similar to their initial study. When calcium supplementation was provided starting 60 min before the exercise bout, serum levels of parathyroid were significantly increased (*p* = 0.05, *p* < 0.001) following exercise [55]. Finally, a 2015 study had 32 competitive female cyclists complete separate 90-min cycling bouts. In one condition, a high-calcium pre-exercise meal was provided and in the other condition, a control meal was provided. When the high-calcium meal was provided, serum levels of bone resorption markers were significantly reduced (*p* < 0.01), suggesting that bone metabolism was favorably managed in response to the prolonged bout of cycling exercise [56]. When viewed collectively, the evidence seems to indicate a benefit of timed calcium supplementation prior to exercise to mitigate exercise-induced disruption to calcium homeostasis.

### Timing strategies for performance and to mitigate adverse events

#### Sodium bicarbonate

Sodium bicarbonate (NaHCO_3_) is an alkalizing agent that has been reported to improve performance by minimizing the development of metabolic acidosis, a key contributor to fatigue during bouts of high-intensity exercise [57] by augmenting the body’s buffering capacity. While several studies have shown conflicting results, multiple studies still show support as an ergogenic aid. For example, a 2012 meta-analysis highlighted multiple studies showing ergogenic outcomes after repeated cycling sprints and submaximal cycling bouts in conjunction with sodium bicarbonate administration [57]. Interestingly, timed administration of sodium bicarbonate may have as much to do with the minimization of gastrointestinal (GI) distress as the promotion of an ergogenic outcome [58]. Furthermore, fear or previous personal experience of GI discomfort from sodium bicarbonate supplementation may increase the avoidance among individuals [59]. Regardless, two studies have suggested that the minimization of GI distress may occur when sodium bicarbonate is consumed for multiple days leading up to an event versus an acute single dose [60, 61]. Further, it has been recommended that ingesting smaller doses of sodium bicarbonate throughout the day, and with food, may also minimize the risk of GI discomfort. Siegler and colleagues [58] explored timing strategies for sodium bicarbonate supplementation and found that pre-exercise timing can favorably impact subsequent reports of GI upset. Using a randomized, counterbalanced, single-arm (no placebo) design, the researchers provided eight male sprinters with 0.3 g/kg sodium bicarbonate at 60, 120, or 180 min prior to repeated bouts of sprinting. While differences in sprinting performance were not detected between treatments, reports of gastrointestinal discomfort were significantly reduced (*p* < 0.05) when the dose was provided 180 min prior to exercise [58]. While the lack of a placebo precluded the ability to discuss any ergogenic outcome, these results are important as many athletes are deterred from using sodium bicarbonate due to the commonly known GI side effects. While further research is required to substantiate the conclusions of this study, it appears that optimal timing of sodium bicarbonate may reduce negative adverse events, which may work towards to improving its attractiveness as an ergogenic aid.

#### Beta-alanine

Beta-alanine is a non-proteogenic amino acid that is produced endogenously in the liver and is also acquired through the consumption of meat and poultry [62]. Beta-alanine has consistently been shown to improve high intensity exercise performance [63] (particularly during high-intensity exercise bouts lasting under 60 s [64]), attenuate neuromuscular fatigue in both men and women [65, 66], and increase resistance training volume by enhancing the buffering capacity of skeletal muscle [67]. Beta-alanine itself does not act as a buffer, but it serves as a rate-limiting substrate in the synthesis of intramuscular carnosine, which contributes at least 7% of the total buffering capacity of skeletal muscle [62]. Similar to sodium bicarbonate, the timing of beta-alanine consumption may minimize known side effects associated with beta-alanine use. Paresthesia [68] or flushing [69], is the most commonly reported side effect with beta-alanine use, which normally occurs when a bolus dose of 800 mg or more is consumed [69]. In this respect, typical beta-alanine supplementation regimens involve dividing the total daily dose (most commonly 6–7 g) into smaller doses (commonly 1.4–1.6 g per dose) to mitigate the paresthesia associated with beta-alanine use [62, 68]. While research at the current time is not available outlining the potential impact of timed delivery of beta-alanine to improve performance, future research involving timing strategies should explore these areas (Table 2).

**Table 2:** Timing implications of chronic micronutrient/supplement administration

Nutrient/Dietary supplement	Mechanism of action	Purported benefit	Recommend dosing protocol	Timing-related references
Documented evidence of timing benefit				
Creatine	↑ PCr ↑ ATP	↑ High intensity exercise capacity ↑ Muscle mass ↑ Strength	Time: 10–12 weeks Absolute Dose: 5 g Relative Dose: 0.1 g/kg body mass	[34–36]
Iron	Oxygen transportation, DNA synthesis, Electron transportation	↑ Aerobic Performance ↑ Oxygen Carrying Capacity	Time: 3–6 weeks Dose: 100 mg/day	[47, 48]
Unexplored, but potential timing effect				
Beta-alanine	↑ Production of carnosine	↑ High intensity exercise ↑ Resistance training volume ↓ Neuromuscular fatigue	Time: 4 x/day Dose: 1.3–1.6 g (6–7 g/day)	[62, 68]
Calcium	Muscular contraction; Blunt ↑ levels of parathyroid hormone	↑ Bone density ↓ Parathyroid hormone levels	Time: 60 min prior to exercise for up to 12 months Dose: 1000 IU/day	[53–55]
Sodium Bicarbonate	↓ Metabolic acidosis	↑ Repeated sprints ↑ High intensity exercise	Time: 1–3 h prior Relative Dose: 0.3 g/kg body mass	[58]

*PCr* Phosphocreatine, *ATP* Adenosine triphosphate, *g* grams, *g/kg* grams per kilogram of body mass, *DNA* Deoxyribonucleic acid, *mg/day* milligrams per day, *h* hours, *min* minutes, *IU* International units

## Conclusions

At the current time, research involving the timing of micronutrients and non-nutrients is in its infancy but will likely be an area of future interest for researchers, coaches, athletes, and the general public. Regardless, preliminary data suggests that micronutrient and non-nutrient timing can improve certain physiological responses which may promote improvements in exercise performance. For example, manipulating the timing of caffeine ingestion either prior to or during endurance cycling has been shown to increase performance outcomes, while other modes of exercise require investigation. Conversely, less information is known regarding the efficacy of other acute timing strategies involving dietary nitrates or nitric oxide precursors such as citrulline malate. Similarly, more information is required to determine the efficacy of timing strategies to maximize the effects of chronic supplement consumption. For example, creatine supplementation has been widely studied, but only within recent years (and only in two studies with different statistical approaches and study populations employed) has a timing question been examined. More information is also required regarding the efficacy of micronutrient timing strategies. Furthermore, not all timing strategies included in this review were shown to directly improve performance, as some investigations indicate that certain supplementation protocols may decrease the incidence of unwanted side effects associated with sodium bicarbonate and beta-alanine consumption. Future research should investigate the efficacy of the included timing strategies in a wider variety of exercise modalities and study participant populations. Future investigators should assess the impact of pre-exercise timing of additional acutely-acting supplements such as citrulline malate, and multi-ingredient pre-workout supplements are also likely candidates for additional investigation. As the body of timing-related research evolves, a greater understanding in this space will help athletes to better refine feeding and supplementation regimens to avoid unnecessary dosing, minimize known side effects, and improve training adaptations and performance.

## Data Availability

Data sharing is not applicable to this article as no datasets were generated or analysed during the current study.

## Abbreviations

Ca^2+^: Calcium
g: Gram
GI: Gastrointestinal
IU: International units
kg: Kilogram
km: Kilometer
km: Kilometers
mg: Milligram
NaHCO_3_: Sodium bicarbonate
NO: Nitric oxide
NO_2_^−^: Nitrite
NO_3_^−^: Dietary nitrate
VO_2_max: Maximal oxygen consumption
VO_2_peak: Peak oxygen consumption
